# Endoscopic transorbital removal of a temporomesial cavernous hemangioma

**DOI:** 10.3171/2025.1.FOCVID24183

**Published:** 2025-04-01

**Authors:** Cesare Zoia, Vittorio Ricciuti, Andrea Montalbetti, Giorgia Piras, Roada Bucpapaj, Elisabetta Peppucci

**Affiliations:** 1 UOC of Neurosurgery, Ospedale Moriggia Pelascini, Gravedona e Uniti, Gravedona;; 2 School of Medicine and Surgery, University of Milano-Bicocca, Monza; and; 3 Department of Neurosurgery, Fondazione IRCCS San Gerardo dei Tintori, Monza, Italy

**Keywords:** endoscopic transorbital approach, ETOA, temporomesial hemangioma, endoscopy

## Abstract

In recent years, endoscopic transorbital approaches have found wider applications and more indications. Their use has been described for resection of epileptogenic pathology in the adult population and for lesions located at the temporal lobe. In this video, the authors describe the case of a 47-year-old woman with a 9-month history of episodes of confusion, altered spatiotemporal awareness, and dizziness, only partially responding to antiepileptic drugs. MRI showed a temporomesial cavernous hemangioma. Endoscopic transorbital resection through a modified superior eyelid approach is shown. Radical resection was obtained without any postoperative complications, and an optimal cosmetic outcome was achieved.

The video can be found here: https://stream.cadmore.media/r10.3171/2025.1.FOCVID24183

## Transcript

This is a case of a 47-year-old woman with no relevant past medical history. Symptoms started in March 2023 with episodes of confusion, altered spatial and temporal awareness, and dizziness. Antiepileptic drugs were started, and further imaging investigation were performed.

### 0:43 MRI Showing the Temporomesial Hemangioma.

As you can see in these images, there is a left mesial temporal lesion that showed signs of recent bleeding, possibly a cavernous angioma. Since the lesion was right behind the orbit, we decided to make a lateral endoscopic transorbital approach.^1^

### 1:17 Rationale of the Technique and Considerations.

The endoscopic transorbital approach has many benefits. First, it allows to reach deep-seated lesions without manipulating the temporal lobe, its eloquent areas, and sylvian vessels. It also avoids corticectomy, which is often needed in microsurgical transcranial techniques and avoids large scalp and temporalis muscle incision. Furthermore, compared to anterior microsurgical techniques, such as lateral canthotomy, it allows to have a better view without bone removal and reconstruction. Finally, this technique has great cosmetic outcomes and less pain for the patient.^2^

The main risks are due to the limited space that makes it difficult for the surgeon to control bleeding or unexpected events during surgery. Also, there is a long learning curve for this technique, which can be a disadvantage in the first surgeries.

### 2:10 Alternative Approaches.

The middle cranial fossa and the temporal lobe have been traditionally reached through open microsurgical transcranial approaches,^3^ and these techniques are valid and used nowadays for the majority of pathologies of these regions. Temporomesial hemangiomas can clearly be reached with these techniques, or via anterior lateral canthotomy as an alternative.^4^ In this case, being right behind the sphenoid wing, the lesion can be resected through an endoscopic transorbital approach, and as anticipated before, this technique can limit potential damage to the brain parenchyma or to the sylvian vessels. Furthermore, cosmetic outcomes are better with the endoscopic approach.

### 2:49 Step-by-Step Technique.

First of all, the patient is in a supine position with the head pinned in a neutral fashion. The registration of the neuronavigation is then performed, and the eyelid and orbit are prepped and draped. The following steps are later described in the video: the skin incision, the periorbital dissection, the meningo-orbital band coagulation and incision, the neuronavigation check, the sphenoid wing drill, the dura opening, the lesion resection, hemostasis, the dural closure, and the subcutaneous and skin closure.

### 3:22 Skin Incision.

The incision is right below the eyebrow, aiming directly at the orbital rim where the orbitalis blends with the frontalis muscle. Once the bone is reached, we proceed with blunt dissection of the periorbita in a craniocaudal and lateromedial fashion.

### 3:34 Surgical Landmarks.

As we can see, early coagulation of a lateral periorbital feeder is necessary, and we can continue with the dissection until we reach an important landmark, which is the meningo-orbital band, that we must coagulate and cut.^5^ This allows us to obtain a better manipulation of the orbit and a larger working space.

### 3:50 Drilling of the Sphenoid Wing.

Once we have reached the posterolateral aspect of the orbit with ease, we introduce the drill, and we can make an opening through the greater sphenoid wing, reaching the middle cranial fossa. We can also use Kerrison rongeur to avoid damaging surrounding structures. And then we refine the opening with the drill once again.

### 4:21 Dura Opening and Intradural Dissection.

After we make sure of the position with the neuronavigation, we open the dura. We introduce cottonoids to be more delicate on the cerebral parenchyma, and we identify where the angioma breaks through the surface.

### 4:49 Removal of the Hemangioma and Hemostasis.

At this point, we use a dynamic retraction using the aspirator. Using the aspirator, grasping forceps, and even a curette, we remove the angioma and the hemosiderin around it. Here you can see the total removal of the hemosiderin with the curette and then with grasping forceps. After we make sure that we have removed the whole lesion, we do hemostasis with a hemostatic agent.

### 6:04 Closure Technique.

We then introduce a synthetic dura. We do a bilayered reconstruction below and above the approximated flaps of the dura. Finally, we only suture the subcutaneous layer of the incision, while the skin is approximated and fixed with synthetic glue in order to obtain a better cosmetic outcome.

### 6:38 Postoperative Outcomes.

After surgery, there wasn’t any new deficit, only little eyelid edema that regressed spontaneously. After neurological follow-up, we were able to discontinue antiepileptic drugs with no recurrence.

### 6:49 Follow-Up.

MRI at 3 months showed complete removal of the hemangioma. Lastly, here is shown the good cosmetic result at follow-up.

## Disclosures

The authors report no conflict of interest concerning the materials or methods used in this study or the findings specified in this publication.

## Author Contributions

Primary surgeon: Zoia. Assistant surgeon: Piras, Peppucci. Editing and drafting the video and abstract: Ricciuti. Critically revising the work: Ricciuti, Zoia, Montalbetti, Bucpapaj. Reviewed submitted version of the work: Ricciuti. Approved the final version of the work on behalf of all authors: Ricciuti. Supervision: Zoia, Bucpapaj.

## Supplemental Information

### Patient Informed Consent

The necessary patient informed consent was obtained in this study.
